# Historical spatio-temporal data on North American radical environmental direct-action events

**DOI:** 10.1016/j.dib.2025.111543

**Published:** 2025-04-03

**Authors:** Zack W. Almquist, Benjamin E. Bagozzi, Daria Blinova

**Affiliations:** University of Washington, Seattle, 98195, WA, USA and University of Delaware, Newark, 19716, DE, USA

**Keywords:** Event data, Radical environmental activism, Animal liberation, Animal rights, Environmentalism, United States, Canada

## Abstract

Social and political event data are widely used in scientific research. However, event data concerning the direct actions of radical environmental groups is comparatively scarce, due in large part to inconsistent news coverage and the clandestine nature of the groups involved. Leveraging original reports maintained by radical environmental groups and their allies, this article codes historical spatio-temporal event data on radical environmental direct-action events in the United States and Canada during a period of heightened prominence in radical environmentalism: 1995-2007. The article's event level data include information on event type, date and geolocation, and the target of each event, as well as the original textual reports of each coded event. This data will facilitate a wide variety of qualitative and quantitative analyses of radical environmental activism, alongside validations of recently developed large language model (LLM) tools for event data extraction. We also offer a separate spatio-temporally aggregated version of these same data. This second dataset is aggregated to the 0.5 × 0.5 decimal-degree spatial grid-year level and adds additional environmental-, environmental group-, and social-correlates. Accordingly, this second dataset will readily enable spatio-temporal statistical analyses of radical environmental direct-action events, their causes, and their determinants—phenomena that have been previously under-explored in large N studies.

Specifications Table

**Table:** 

Subject	Sociology
Specific subject area	Radical environmental activism, animal rights, environmental sociology, and environmental politics.
Type of data	Table, Raw, Filtered, Processed
Data collection	A series of radical environmental group magazine PDFs were collected. Sections of these PDFs included past environmental direct action event textual summaries for 1995-2005, and all textual content was extracted via optical character recognition and/or manual transcription. Additional direct action event records for 2002-2007 were webscraped from a radical environmental group-maintained website in plain text format. A series of automated tools and manual coding steps then extracted (coded) event characteristics and deduplicated events. Code was then used to aggregate and merge additional variables to aggregated versions of these coded data.
Data source location	Data were primarily extracted from the 30-issue “No Compromise” magazine, which referred to itself as the “Militant, Direct Action Newsmagazine of Grassroots Animal Liberationists & Their Supporters”. Supplemental data was then collected from the (now defunct) BITE BACK website that was maintained for a period of time following the dissolution of No Compromise.
Data accessibility	Raw and aggregated versions of these data are publicly and anonymously available at the following Harvard Dataverse Repository: Repository name: ``Radical Animal Liberation Movement (RALM) Event Data'' Data identification number: https://doi.org/10.7910/DVN/5V1JU8 Direct URL to data: https://dataverse.harvard.edu/dataset.xhtml?persistentId=doi:10.7910/DVN/5V1JU8
Related research article	None

## 1 Value of the data

• These fine-grained historical event data on the North American radical animal liberation movement (RALM) are novel in terms of overall coverage, direction-action target details, details on direction-action event type(s), and information on animal species released.
• The spatio(temporal) information available for each associated event, and a separately provided spatio-temporally aggregated dataset with covariates, will together enable future researchers to undertake previously unachievable spatio-temporal statistical analyses of RALM direction-action drivers and consequences.
• The ontology developed for RALM direct-action coding can serve as a future template for subsequent coding projects associated with radical environmentalist groups and their direct-actions.
• The data's human-labeled event characteristics and accompanying original source texts secondarily offer a unique set of full texts and human labels for future large language model (LLM) development and validation in the socio-political event data extraction area.

## 2 Background

Radical environmental activism is an important area of study in the fields of political science, sociology, criminology, psychology, and legal studies, among others. However, quantitative research into such activism remains scarce [[1], [2]]. This is surprising given that quantitative studies of broader forms of socio-political unrest in sociology and political science are experiencing a renaissance thanks in part to recent developments in fine-grained, geolocated socio-political event data [[3], [4], [5]]. One reason for the absence of detailed environmental direct-action events within such socio-political event data is incomplete reporting on these oftentimes ‘low level’ direct-action events within the traditional news(wire) media that socio-political event datasets typically rely upon for coding. This article addresses this challenge by identifying and human-coding a set of comprehensive textual records of low-level direct-action events for the radical animal liberation movement (RALM) across the United States (U.S.) and Canada over a 12-year period. These textual records are taken directly from records curated by the RALM and its supporters, ensuring unprecedented detail and coverage in the coding of socio-political event data pertaining to the North American RALM and their direct-actions.

## 3 Data Description

There are two datasets associated with this article. The first contains raw, event level data on environmental direct actions and associated textual content. The second is a spatio-temporally aggregated event dataset measured at the grid cell-year level. This section describes each dataset in turn.

The article's associated raw radical animal liberation movement (RALM) dataset is named RALMevent.csv. This dataset is at the individual event level. Each row corresponds to a discrete atomic event and each column denotes an event characteristic or original coding source characteristic. The primary years of coverage are 1995-2007. However, a small number of 1994 events were available in initial source materials. These initial 1994 events are included in RALMevent.csv for reference, although the coverage of direct-action events in 1994 is not comprehensive. The dataset specifically includes the event-level variables outlined in Table 1.

**Table 1:** Variables and variable descriptions for raw RALM Event-Level Dataset (RALMevent.csv).

Variable Name	Description
issue	The No Compromise issue number of the event's original textual summary. If the event was instead coded from the BITE BACK website, issue is left blank
month	The numeric month of the recorded event
day	The numeric day of the recorded event
year	The numeric year of the recorded event
city	The city location of the recorded event, using standardized free-form text
province.state	The Canadian Province or U.S. State of the recorded event
named.location	The free-form text of any specific business or non-commercial location that was reported as being the target of the event
location.type	A named.location category: Business, Government, Police, Airport/Airlines, Diplomatic, Educational Institution, Food/Water Supply, Media, Maritime, NGO, Private Citizens, Telecommunication, Transportation, Utilities, Organized Gathering, Wild Animal Trap/Sensor, Natural Environment, Unknown, or Other
latitude	The latitude coordinates of the city associated with the event
longitude	The longitude coordinates of the city associated with the event
category.a	The event's primary event classification, based on 1: Release, 2: Assault, 3: Damage, 4: Arson, 5: Harassment, 6: Threat, 7: Theft. If only one event-type appeared for an atomic event, this is the only ``Category'' field recorded
category.b	The event's second event classification, based on: 1: Release, 2: Assault, 3: Damage, 4: Arson, 5: Harassment, 6: Threat, 7: Theft. Only coded if at least two event-types appeared for an atomic event.
category.c	The event's second event classification, based on: 1: Release, 2: Assault, 3: Damage, 4: Arson, 5: Harassment, 6: Threat, 7: Theft. Only coded if three event-types appeared for an atomic event.
animal.1	If an event category was of animal liberation, free-form text of the type of animal liberated, such as, for example, “Mink”, “Chicken”, “Fox”, “Dog”, or “Rabbit”
animal.2	If an event category was animal liberation and more than one species was liberated, the free-form text of the second type of animal liberated (see animal.1 for examples)
animal.3	If an event category was animal liberation and more than two species were liberated, the free-form text of the third(+) type of animal liberated (see animal.1 for examples)
text	The transcribed textual summary for a given event-entry, as appearing in No Compromise or BITE BACK

This event-level dataset directly enables several types of analysis. First, it facilitates qualitative identification of, and investigations into, individual direct-actions or subsets of direct-actions (e.g., according to a specific location, type, or time-period). Second, this specific event-level dataset flexibly allows quantitative researchers to subset and/or aggregate all associated RALM direct-action events to a researcher's own desired level of specificity. This, in turn, ensures that the data coded under this project can be readily combined with any additional researcher-developed datasets and structures, as based upon included identifier variables in RALMevent.csv such as month, day, year, province.state, city, latitude, or longitude. Third, given the growing importance of large language models (LLMs) to event data extraction [5], the RALMevent.csv dataset's human-extracted labels for event type(s), named location, geolocation, and animal(s) liberated—along with the inclusion of the full source texts that were used to code these labels—provide gold standard training and/or validation set for future LLM development and/or validation in this area. Note that most socio-political event datasets rely upon copyrighted news texts that are not provided within released event datasets, thus limiting the usefulness of such datasets for secondary LLM training and validation. Given the non-print news origins of the source texts used for the RALM data described here, all corresponding texts are included within RALMevent.csv, thereby ensuring that this dataset is not limited in this manner.

**Fig. 1:**
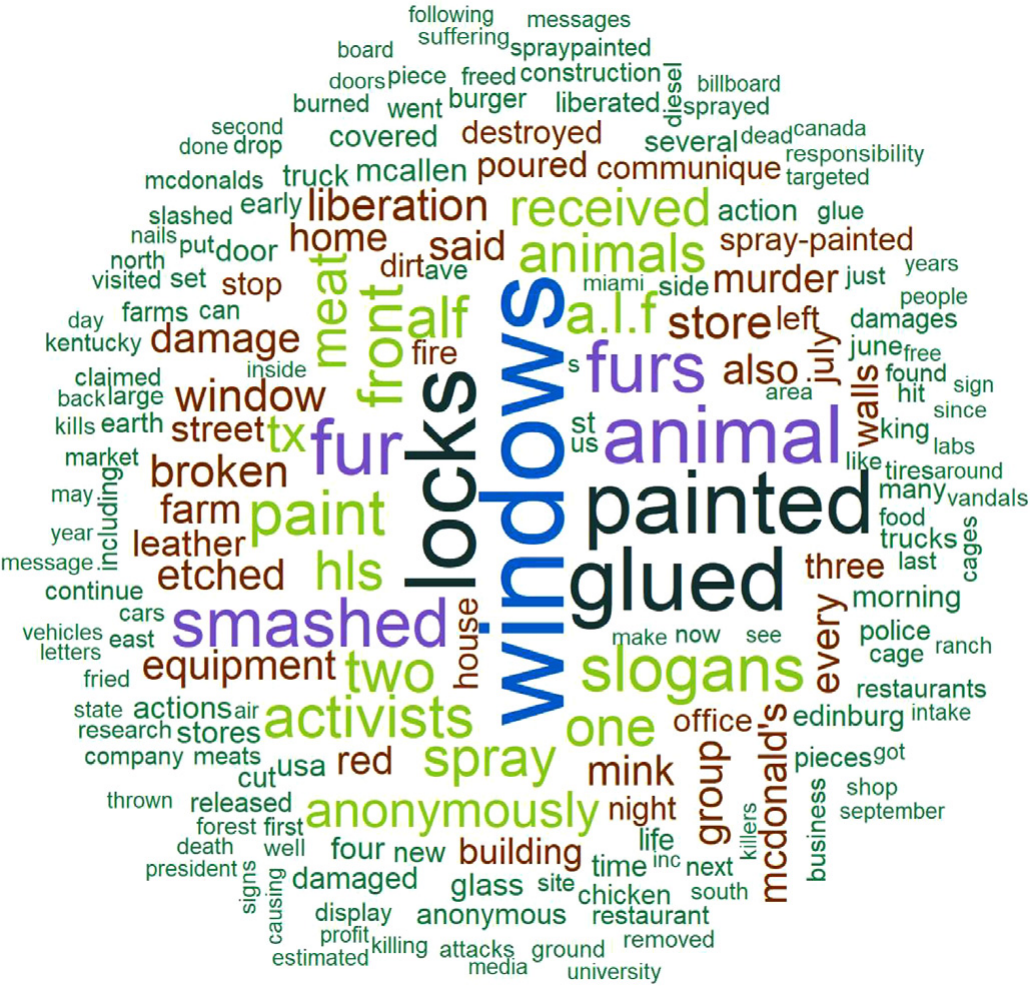
Word cloud of textual descriptions in RALMevent.csv.

**Fig. 2:**
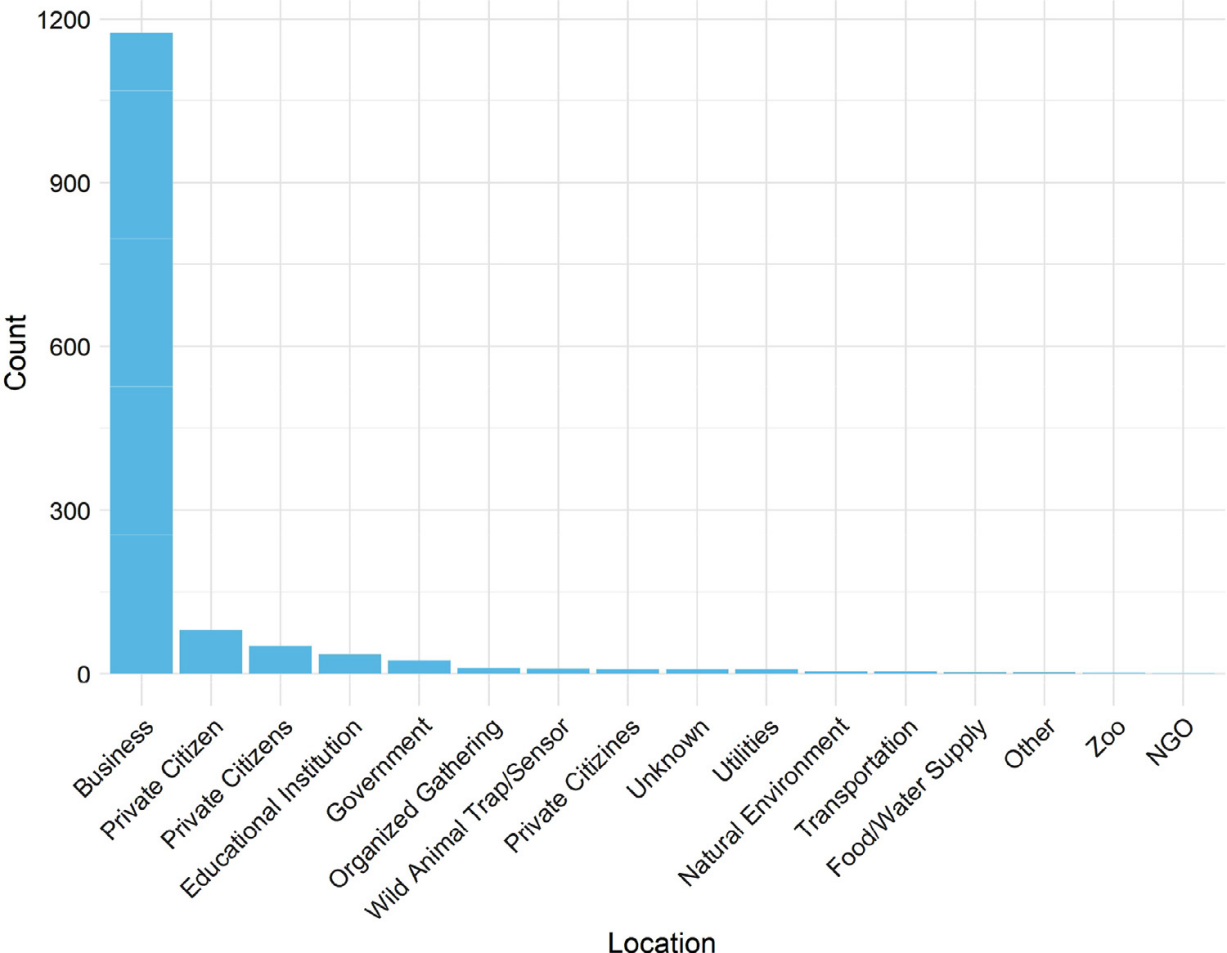
Frequencies of each RALM Event's named.location Type(s) in RALMevent.csv.

**Fig. 3:**
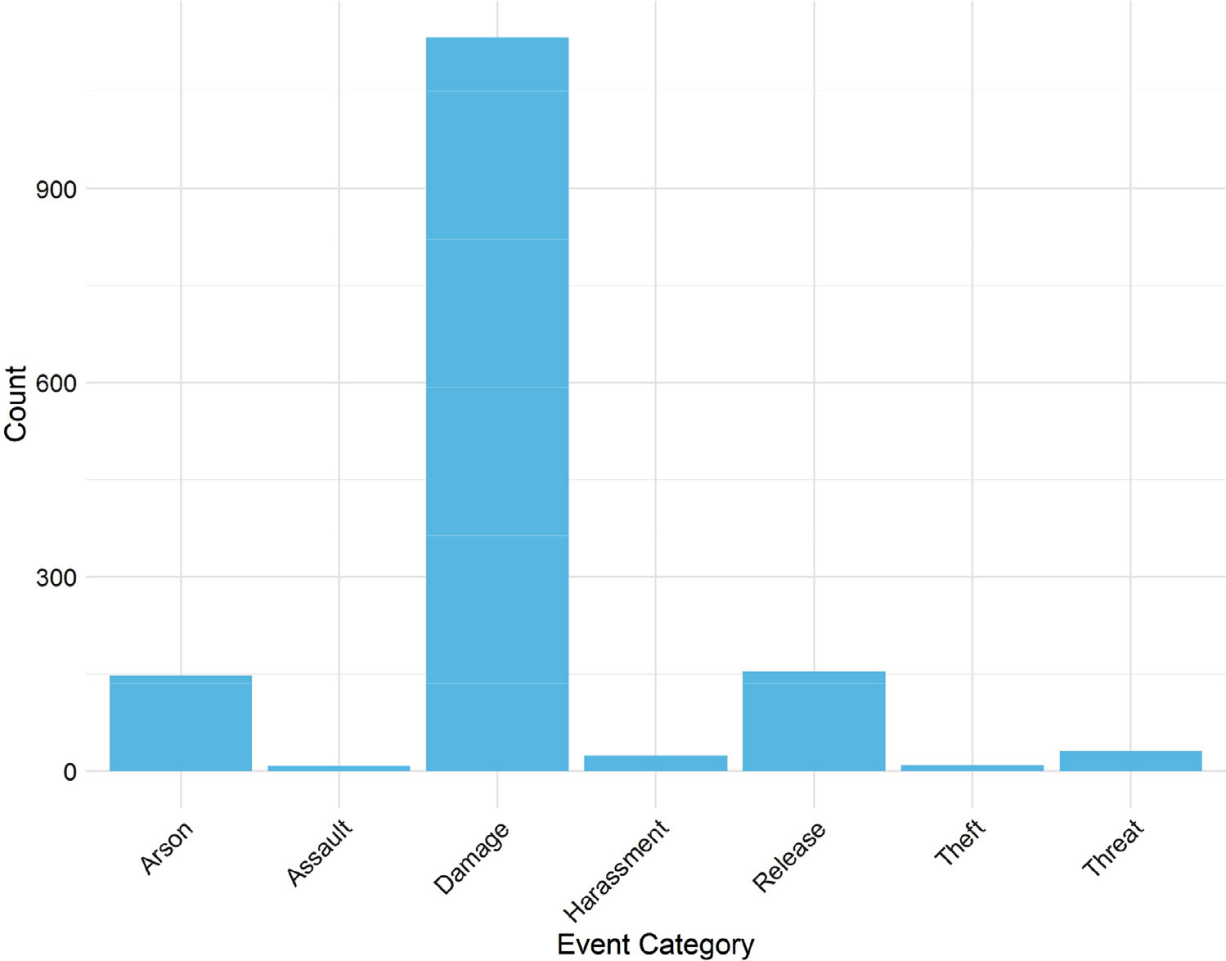
Frequencies of each RALM Event's Event Category in RALMevent.csv.

**Fig. 4:**
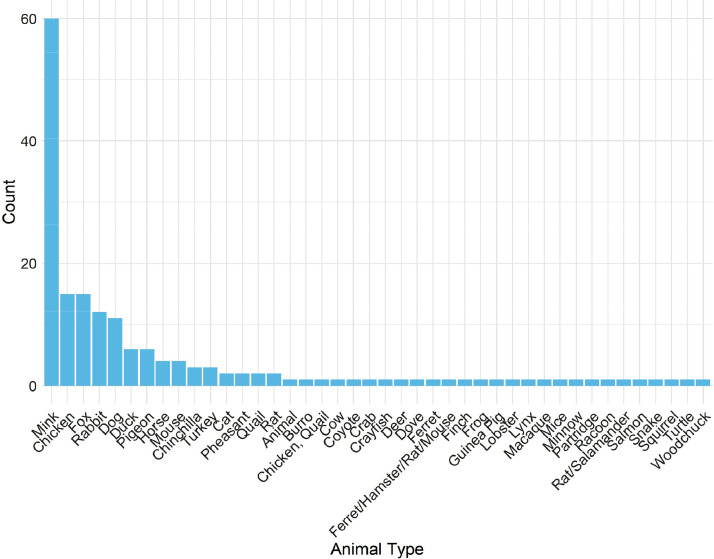
Frequencies of each RALM release events’ released animal types in RALMevent.csv.

The word cloud in Fig. 1 provides an illustration of the textual summaries in RALMevent.csv. This word cloud was created after omitting English-language stopwords, symbols, and numbers, and after converting all remaining text to lower-case. One can observe in Fig. 1 that the most common words across these textual summaries focus on active acts of damage, including the gluing of locks (‘locks’, ‘glued’), the damage of windows (‘smashed’, ‘windows’), and associated targets (‘fur’, ‘furs’, ‘store’). The bar plots in Fig. 2, Fig. 3, Fig. 4 in turn illustrate the relative frequencies of named.location types, event categories, and animals liberated within the RALMevent.csv data. Reinforcing the above observations, one can note in Fig. 2 that the most frequent location targets in the RALM data are businesses, followed by private citizens, educational institutions, and government targets.

Likewise, Fig. 3 demonstrates that the most frequent direct-action events in the RALM data pertain to (property) damage, with arson and animal release efforts also showing sizable numbers in terms of instances. By comparison, thefts and assaults were far less frequent across the RALM's North American activities for period of interest. Fig. 4—which presents the frequencies of different animal types released or taken by the RALM within release events—in turn demonstrates that for those RALM events involving animal releases, minks were by far the most frequent target.

**Fig. 5:**
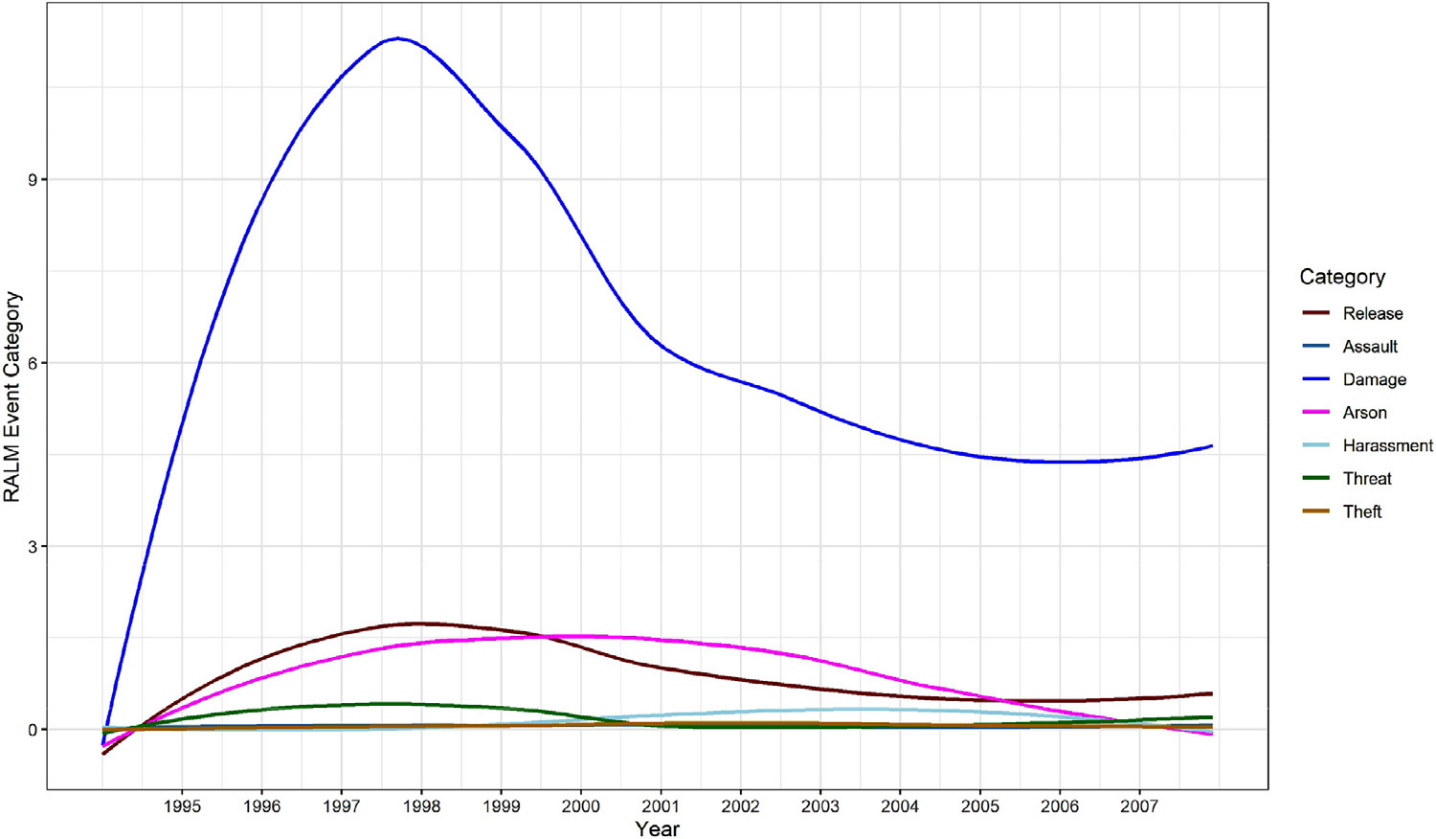
Temporal intensities of RALM events in RALMevent.csv, by event category.

Finally, the generalized additive model (GAM)-smoothed temporal plots in Fig. 5 illustrate the changing frequency of RALM direct action events across the 1994-2007 period. These plots were created after summing all events with available information to the monthly level for each specific event category in RALMevent.csv. Recall here that, as noted previously, the 1994 events included in RALMevent.csv are incomplete, ensuring that the plotted totals for the initial year in Fig. 5 likely underreport true events. As was the case for Fig. 3, one can notice here again that events involving (property) damage were by far the most frequent type of direction-action undertaken by the RALM. Yet, one can further observe in Fig. 5 that these property damage-oriented events peaked in 1998, declining thereafter. Animal releases, arsons, and threats each exhibit similar trends of increase followed by declines over this period, albeit with different peaks.

This article's second provided dataset is a spatio-temporally aggregated version of the raw dataset discussed above. It is intended to more readily enable large N statistical analyses. It is named RALMaggregated.csv and is constructed to only include events and years beginning in 1995 (thus omitting the small number of 1994 events mentioned above). For this spatio-temporally aggregated dataset, the individual events within RALMevent.csv were spatially aggregated (i.e., summed) to the 0.5 × 0.5 decimal degree grid cell-level for the US and Canada and were temporally aggregated (i.e., summed) to the yearly level for 1995-2007. The grid cells used for this aggregation were taken from PRIO-GRID [6], which is a commonly used and widely understood spatial data structure within political science, conflict studies, and related fields such as sociology. A wide array of additional grid(-year) variables were then added to the aggregated RALM data, including several from PRIO-GRID itself. The RALMaggregated.csv and its associated variables and structure is discussed below.

We present and discuss the variables in RALMaggregated.csv in three parts. First, a core subset of variables associated with RALMaggregated.csv is presented in Table 2. Here one can note several identifier variables pertaining to each grid cell's PRIO-GRID identifier (gid), the latitude and longitude coordinates of its grid cell (gidlatitude and gidlongitude), as well as its associated temporal (year) and overarching spatial (country, gwno, or provincestate) association. Alongside these identifiers, variables are included for the total number of RALM direct-action incidents in a particular grid cell-year (across all categories), as well as potential correlates such as measures of environmental disaster and of the total number of active RALM groups in a particular grid cell-year.

**Table 2:** Core variables included in Aggregated RALM Dataset (RALMaggregated.csv).

Variable Name	Description	Source
gid	The individual grid (spatial unit)’s PRIO-GRID ID	[6]
gidlatitude	The latitude coordinate of the PRIO-GRID centroid	[6]
gidlongitude	The longitude coordinate of the PRIO-GRID centroid	[6]
country	The grid cell's primary country (U.S. or Canada)	Authors
provincestate	The grid-cell's primary Canadian Province or U.S. State	Authors
year	The associated year	Authors
incidents	The number of RALM events for that PRIO-GRID-year	Authors
groups	The number of RALM groups active in that PRIO-GRID-year	Authors
disastercost	Total cost (in millions, $U.S.) of disasters in that PRIO GRID-year	[7]
disasterfatality	Total fatalities associated with disasters in that PRIO GRID-year	[7]
disasterevent	Whether or not a PRIO GRID-year saw at least one disaster	[7]

**Fig. 6:**
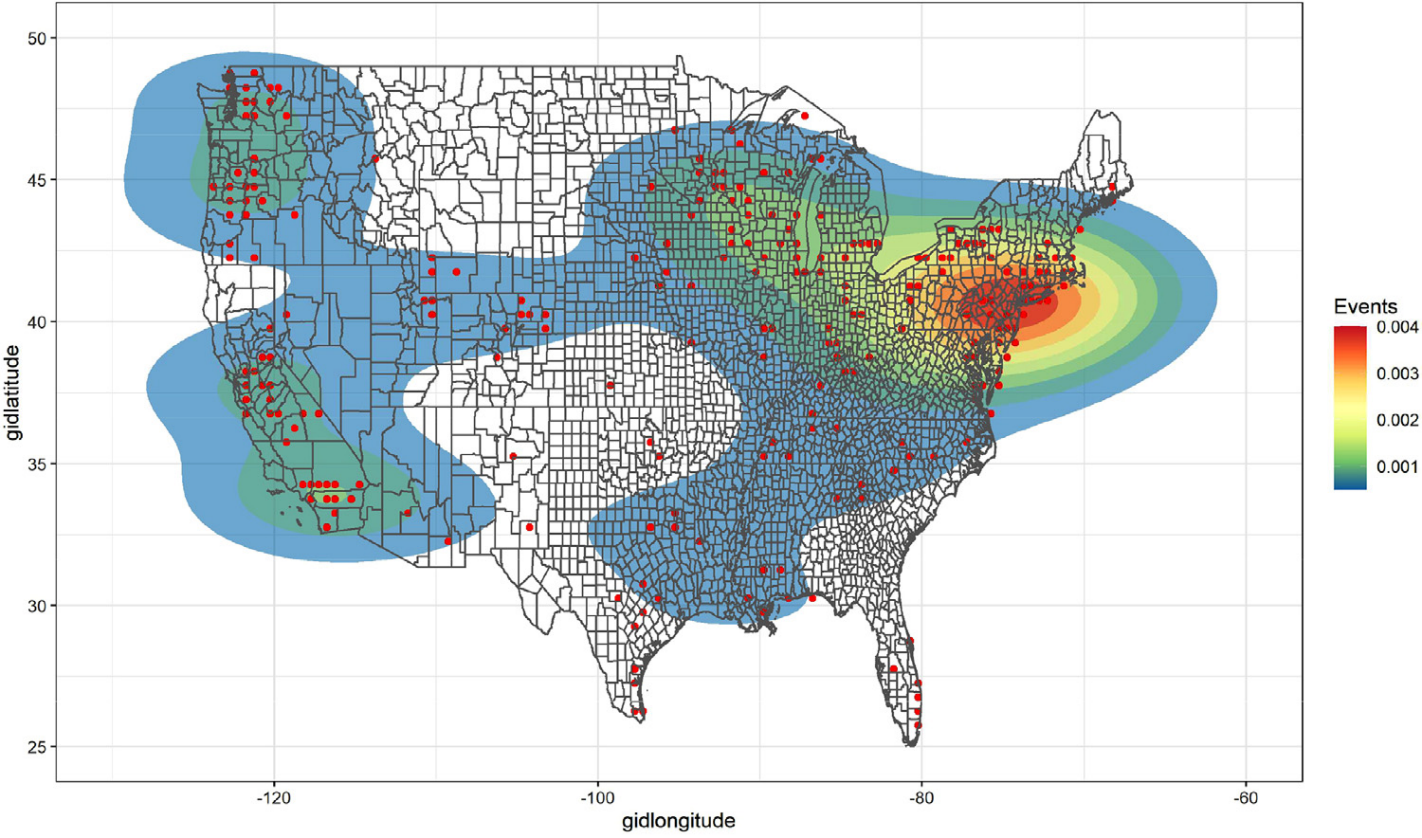
Heat map of continental U.S. events in RALMaggregated.csv.

**Fig. 7:**
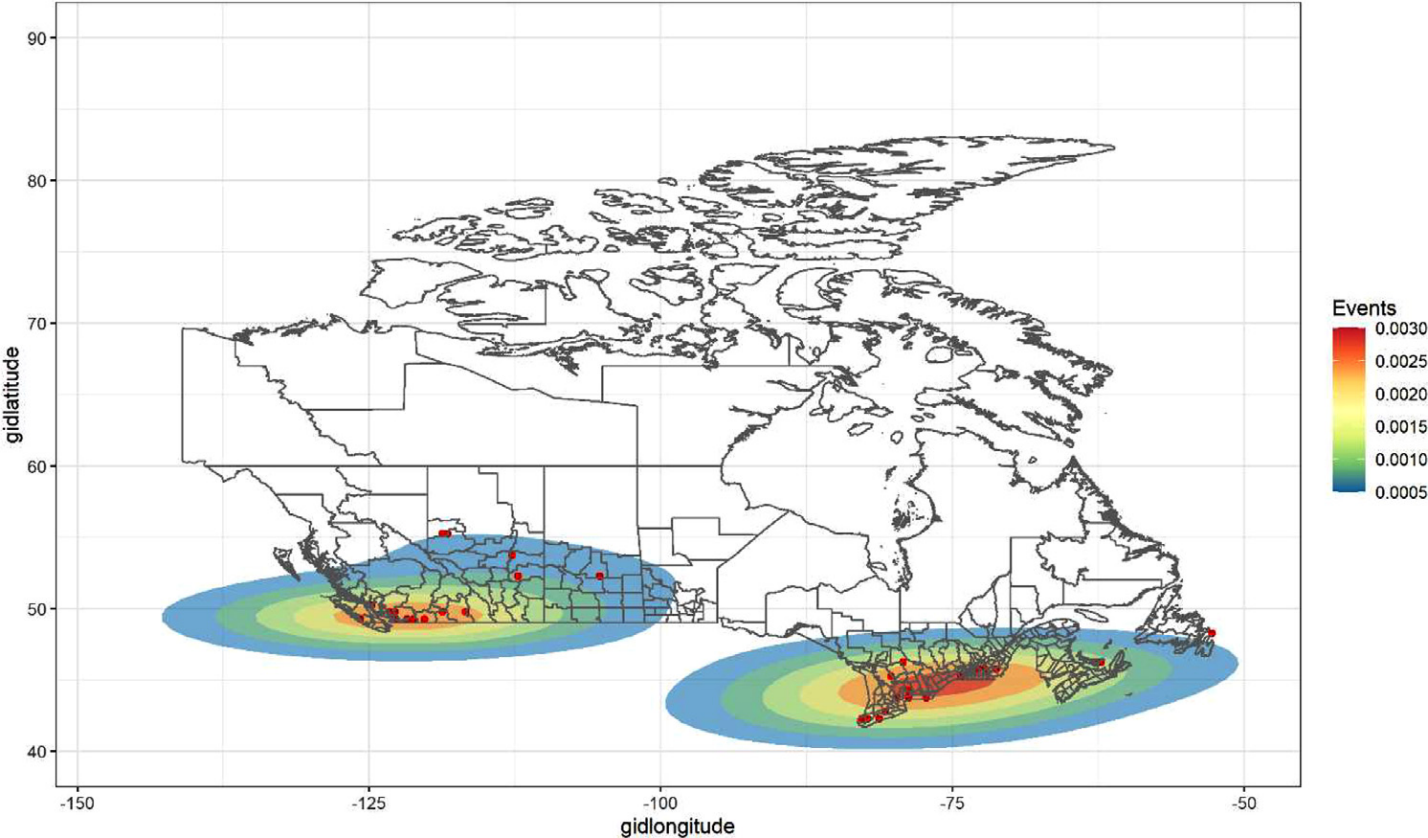
Heat map of continental Canada events in RALMaggregated.csv.

Leveraging this aggregated data, we present a pair of heat maps of all radical environmental events in RALMaggregated.csv. To illustrate the spatial variation available in this dataset, the dataset's recorded events across the years 1995-2007 were plotted separately on heat maps of the continental U.S. and Canada in Figs. 6 and 7 below. The scale (and legend) in each plot corresponds to the density of events within each corresponding shaded local area, based upon the number of points in that area divided by the corresponding local area's size. Local area size in this case will vary but should fall between roughly 55km x 55km and 111km x 111km depending on the specific coordinates of a particular area, after accounting for the (0.5 × 0.5 decimal degree) size of the underlying PRIO-GRID cells in the data and the default kernel bandwidth of the heat map's density plotting function. Note furthermore that while RALMaggregated.csv includes all events (and additional variables) across both the US and Canada Alaska and Hawaii were omitted from these plots. The U.S. and Canada were also split up across Figs. 6 and 7 for ease in visualization.

As one can see in Figs. 6 and 7, the incidents data in RALMaggregated.csv are spatially sparse. Even so, one can clearly observe patterns of spatial clustering (i) in the U.S. Mid-Atlantic, Midwest, California, and Pacific Northwest (Fig. 6) and (ii) in Southern British Colombia and the Quebec City–Windsor Corridor (Fig. 7). Furthermore, as alluded to further above, the PRIO-GRID spatial structure is now commonplace in analyses of social unrest and related events at this level of spatial(-temporal) resolution [[8], [9], [10]]. Thus, the structure and units of analysis in RALMaggregated.csv will be familiar and usable to researchers interested in conducting spatio(temporal) statistical analyses of RALM events.

Researchers analyzing these aggregated data can utilize the included gid, gidlatitude, gidlongitude, country, provincestate, and/or year variables to merge additional RALM event correlates to this dataset prior to analyses. At the same time, RALMaggregated.csv also includes a number of already merged covariates to more readily facilitate such analyses. A first set of additional covariates includes the three disaster-specific variables highlighted in Table 2 (i.e., disastercost, disasterfatality, and disasterevent). Together these covariates provide grid cell-year measures of major environmental (and primarily energy-related) disasters or their impacts in the countries, grid-cells, and years considered, as coded by the current article's authors from the materials in [7].

Two additional sets of added covariates within the second dataset (RALMaggregated.csv) discussed here are detailed in Tables 3 and 4 below. First, Table 3 provides a set of variables reflecting general socio-political event interactions involving a country's (i.e., the U.S.’ or Canada's, depending on the grid cell) civilians and/or government actors, as aggregated from [4]. These measures represent directed quad-category event counts of verbal and material cooperative and conflictive interactions for relevant actor pairings and can be seen as measures of broader (i.e., not explicitly environmental) social or socio-political tensions within each grid cell-year observation. Using the CAMEO event ontology [11], these quad categories are well established [[12], [13], [14]] divisions of CAMEO's two-digit event categories into verbal and material cooperation and conflict events based upon the following categorizations:

• Verbal Conflict: 09: Investigate; 10: Demand; 11: Disapprove; 12: Reject; 13: Threaten; 16: Reduce Relations
• Verbal Cooperation: 03: Express Intent to Cooperate; 04: Consult; 05: Engage in Diplomatic Cooperation
• Material Conflict: 14: Protest; 15: Exhibit Force Posture; 17: Coerce; 18: Assault; 19: Fight; 20: Use Unconventional Mass Violence
• Material Cooperation: 06: Engage in Material Cooperation; 07: Provide Aid; 08: Yield

**Table 3:** General event data variables included in aggregated RALM Dataset (RALMaggregated.csv).

Variable Name	Variable Description
govcitverbconf	Number of government -> civilian verbal conflict events
govcitmatconf	Number of government -> civilian material conflict events
govcitverbcoop	Number of government -> civilian verbal cooperation events
govcitmatcoop	Number of government -> civilian material cooperation events
citgovverbconf	Number of civilian -> government verbal conflict events
citgovmatconf	Number of civilian -> government material conflict events
citgovverbcoop	Number of civilian -> government verbal cooperation events
citgovmatcoop	Number of civilian -> government material cooperation events
citanyverbconf	Number of civilian -> any verbal conflict events
citanymatconf	Number of civilian -> any material conflict events
citanyverbcoop	Number of civilian -> any verbal cooperation events
cityanymatcoop	Number of civilian -> any material cooperation events

**Table 4:** PRIO-GRID variables included in aggregated RALM Dataset (RALMaggregated.csv).

Variable Name	Description
bdist3	Distance (km) of cell to nearest border of nearest land-contiguous neighboring country
capdist	Distance (km) to capital city in cell's overarching country
droughtyr_spi	Proportion of year's months in consecutive drought (i.e., the number of longest consecutive drought months in that year divided by 12). A drought month is determined by a (greater than -1.5) deviation from long-term normal rainfall for a particular month and location
excluded	Number of excluded (discriminated or powerless) groups
gcp_mer	Gross cell product in US dollars
gwno	Gleditsch & Ward country code
pop_gpw_sum	Total population in grid cell
prec_gpcp	Total annual precipitation (millimeters)
agri_gc	Agricultural land coverage in cell
imr_mean	Average infant mortality rate in grid cell
landarea	Total land-covered area in grid cell (square kilometers)
ttime_mean	Average travel time (in minutes, by land-transportation, to nearest major city) within grid cell
Urban_gc	Urban land coverage in cell, in terms of the urban percentage of all land area for that cell

Next, Table 4 presents the additional PRIO-GRID variables taken from [4] that are included in RALMaggregated.csv. These variables allow one to account for additional grid(-year)-level factors related to geography (e.g., distance to borders or capital cities), the environment (e.g., precipitation or agricultural ground cover), or society (e.g., population and infant mortality rates). More detailed descriptions of each variable—beyond what is provided in Table 4—can be found in [6] and its accompanying materials.

## 4 Experimental design, materials and methods

The original source materials used for coding each RALM direct action event include (i) printed sections found within the 30-issue magazine known as No Compromise and (ii) webscraped summaries from a now defunct website known as BITE BACK (https://tinyurl.com/nhf62rhn).^1^

**Fig. 8:**
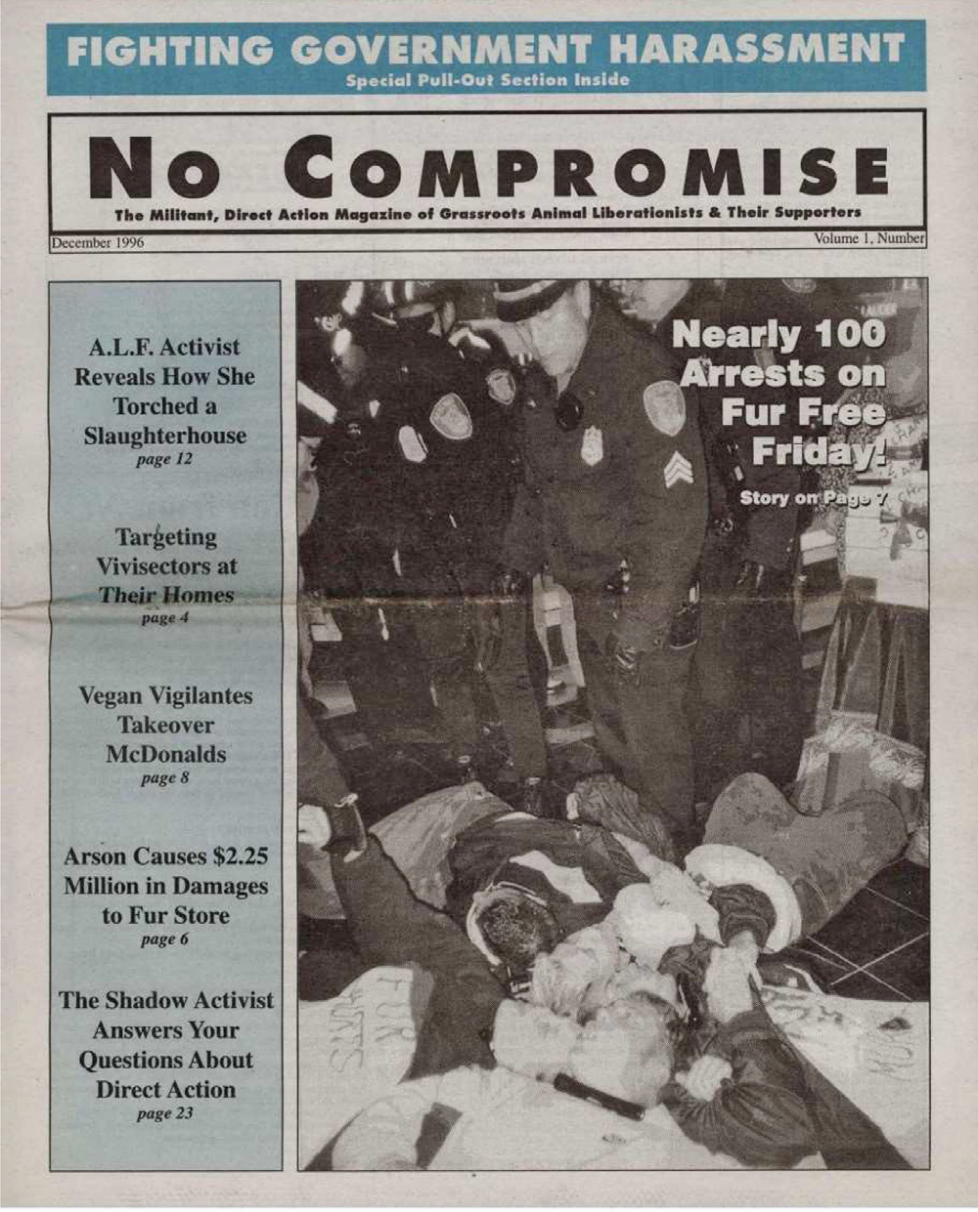
No compromise, Issue 5.

No Compromise was a radical animal rights magazine (‘zine) that saw 30 issues printed from 1996 to 2006. Fig. 8 presents an example image of No Compromise from Issue 5. The ‘zine described itself to varying degrees (depending on the issue) as the voice of the North American animal liberation movement, including that of the Animal Liberation Front (ALF). Issues 1-27 of No Compromise each include a section summarizing the direct-actions perpetrated by radical environmental animal rights groups since the previous issue. These direct-actions were generally submitted anonymously in writing to No Compromise by the individual or group that perpetrated the direct-action. An example of a submitted direction-action summary is as follows:

• “08/02/1995; New York: Meat trucks at Hofmann's Sausage Company in DeWitt had their tires slashed. Damages cost $3,500.”

All 30 issues of No Compromise were downloaded from online archives of radical environmentalist publications [15]. The relevant passages of direct-action events in these issues were converted to plain text through a combination of optical character recognition (OCR) and manual transcription. Towards the very end of No Compromise's publication, the ‘zine became more retrospective and potentially less comprehensive in its coverage of individual direct-action events. To ensure complete coverage through 2007 (a period encompassing several of the additional covariates included in RALMaggregated.csv,) comparable event summaries were webscraped from an online successor to No Compromise known as BITE BACK (https://tinyurl.com/nhf62rhn)^2^ for the period 2002-2007. Like No Compromise, this website is no longer active.

All transcribed and scraped events across those included in No Compromise and BITE BACK were first deduplicated by manually comparing each event obtained from the latter source to those obtained in No Compromise for the years 2002-2005. After deduplication, all unique event summaries were combined into a spreadsheet for manual coding across the variables described in RALMevent.csv and Table 1. The spreadsheet initially included these textual summaries along with an indication (in a separate column) of the No Compromise issue that each summary was drawn from, which was left blank for summaries that were instead drawn from BITE BACK. The variable coding details, instructions, and process were as follows.

**Recording the Event's Basic Spatio-Temporal Information**: In the header of each event entry in No Compromise or BITE BACK, there is typically a bolded Date - City label that precedes the event itself. The Date component is usually in MM/DD/YY format. Using this information, a human coder (also an author on this article) entered information into a set of “Month,” “Day,” and “Year” columns for that event within the coding spreadsheet. In cases where month, day, or year information was missing, the associated variable(s) was (were) left blank.

Next, the No Compromise ‘zine's overarching content or the subsequently transcribed/scraped event summaries were used to code each direction-action event's geographic location information according to separate variables for the event's country, province/state, and city. For the earlier issues of No Compromise, relevant information was included in a COUNTRY header that contained a list of city-specific (and occasionally provincestate-specific) events underneath it. In later issues and in the scraped texts, there was often instead a (bolded) header for each individual event that contains “City, Country” information; or that information was contained within the event summary itself. Where a city or province/state was not provided for a given event, that event's entry on the corresponding city and/or provincestate variable(s) was (were) left blank. In cases where an event's sub-country or sub-province/state geographic information was imprecise (e.g., ‘near Toronto’), that description was transcribed into its most appropriate column (e.g., City) for use in subsequent geolocation.

**Separating Multi-Event Records**: Each transcribed event summary was then manually reviewed to determine whether it describes (i) a single event (potentially involving multiple event categories or tactics) or (ii) multiple distinct events at multiple distinct locations. An example of a summary describing a single event would be:

• “9/01/96 - Morris Kaye and Sons Fur shop had locks glued and slogans painted.”

An example of summary describing multiple (in this case, three) distinct events is as follows:

• “6/26/96 – Mercer Island, WA: A McDonald's on 77th Ave. S.E. was hit with a brick through the window and ``Meat Is Murder'' and ``A.L.F.'' painted on the building's north and south sides. Baskin Robbins which had locks glued and animal liberation slogans painted on the walls. Subway Subs also had a window broken and graffiti painted on a rear wall.”

If an event entry described only a single event, no further adjustments were made under the present step. However, if an event entry described multiple events, a separate row was created for each distinct event, with the previously extracted summary information and associated date/location information repeated within each of those rows. This ensures that each record in the subsequent coding step and final RALMevent.csv data corresponds to a single atomic event record.

**Named Location, Location Type, and Geolocation**: Several location-specific variables were manually coded based upon each final event summary or previously extracted city variable. First, the coder fully transcribed the named location (i.e., target) of an event—as it appeared within the event's summary—within a separate named.location column in the coding spreadsheet. For example, in the multiple event example in the section immediately above, the named.locations for each event would be ``McDonalds,'' ``Baskin Robbins,'' and ``Subway.'' These extracted named.location entries are based strictly off of the descriptions provided in each event summary and hence range in specificity, in some cases conveying a very general location (e.g., “shop”) or in other cases a more specific location (e.g., “McDonalds”). Based on named.location, and auxiliary information in an event's summary text, the coder then assigned a categorical variable value for that location's location.type in a separate spreadsheet column. Possible location.types were: business, government, private citizen, educational institution, organized gathering, wild animal trap/sensor, utilities, natural environment, transportation, food/water supply, zoo, ngo, other, or unknown.

For these location.type codings, business was applied to only pertain to the targeting of physical businesses, including animal farms. ‘Educational institution’ encompasses schools, colleges, universities, and university research labs/stations. ‘Ngo’ corresponds to non-government organizations, non-profits, mainstream environmental organizations, and similar interest groups. The ‘private citizen’ location.type was coded to correspond to any individual whose home, personal property, or person was targeted by a direct-action, even if they are the employee of a company that itself is the broader intended RALM target. ‘Utilities’ includes energy (production or supply) utilities/infrastructure, water and sewer infrastructure, telecommunications infrastructure (wireless towers, phone lines, cable lines, satellites, etc.), and/or the entities overseeing these industries and infrastructure. ‘Transportation’ corresponds to the targeting of public roads or other transportation infrastructure (e.g., trains, railway lines, ferries, ferry ports). ‘Organized gatherings’ are public auctions, conferences, meetings, hunts, and related events, no matter the identities of attendees (e.g., as NGO representatives, private citizens, government employees, and so on). The ‘wild animal trap/sensor’ category corresponds to any hunting equipment or animal monitoring equipment, including traps set for both hunting and fishing, research-oriented animal monitoring equipment, and hunting tree stands. ‘Natural environment’ pertains to any acts targeting actual natural spaces, landmarks, or flora, such as tree-spiking. ‘Other’ was reserved for cases where the target did not fit any of the above categories whereas ‘unknown’ was only applied in the very rare instances where a target was not described.

Finally, the city level information that was previously extracted was utilized to assign a separate longitude and latitude coordinate for each event with sufficient geographic location information. To do so, the city itself was queried in geonames.org, and the appropriate city-level latitude-longitude coordinates were then transcribed manually into the coding spreadsheet. Geonames.org is a common open-source geolocation resource used for geolocating socio-political events [5,13,16].

**Event Categories**: To code each entry's event category or categories, the full event summary text was first re-read for coding. Even for individual atomic events, an event may have multiple categories if different tactics were used in the same action. An example of an atomic event with one category is:

• “7/11/00: Kerrville TX – “Meat is Murder” written in Taco Casa bathroom.”

An example of an atomic event with two categories is:

• “AUG 13/95: Annedale: 1 coyote released from Davidson Fur Farm, a sign was spray painted.”

In light of this, three separate event category variable columns were created in the coding spreadsheet. The first was used as the default and primary event category. The second and third event category columns were only coded in instances where an atomic event had multiple direct-action tactics. For each atomic event, and for each relevant action category for that event, coding then entailed the entry of a numeric action category code based on the following coding ontology (Table 5):

**Table 5:** Coding details for RALM direct action categories.

Category Code	Category Name	Coding Description
1	Release	The freeing, liberating, releasing, rescuing, or stealing of (an) animal(s)
2	Assault	Attempted or successful physical assault of a human, with evidence of intention. Examples include letter bombs/bombings, letters including razor blades, and food/water poisonings (excluding hoax poisonings)
3	Damage	Damage to physical property aside from arson. This includes lock gluing, instances where windows are smashed or shot out, tree spiking, survey stake removal/destruction, wire-cutting, etching, paint(-stripper) or tar bombings, tagging/spray painting (of logos or slogans/statements), spray-painting (ambiguous), tire slashing, or the addition of corrosive materials put to vehicle/car engines/building materials, and so on
4	Arson	Burning/arson of buildings, signs, or cars; the use of incendiary devices, firebombing, explosives/explosions, Molotov cocktails, and so on. This also includes attempted uses of these devices that failed to detonate
5	Harassment	The use of (primarily electronic) means of harassment absent a direct threat. This includes ambiguous/prank phone calls, illegal purchases with others' credit cards, DDOS (online) attacks, ordering excessive numbers of items (e.g., magazines/pizzas/taxis) to residences, emailing individuals' friends/neighbors with compromising information, and physically mailing information to individuals or friends/neighbors
6	Threat	Threats that are more direct and explicit than general harassment, typically targeting a specific individual or entity. This includes bomb threats, fake bombs placed at locations, similar hoax devices placed at locations, fake poisoning threats made to communities or the general public, written threats or ``warnings'' made during property defacement (or in letter/note form), and specific effigies left at residences
7	Theft	Theft of physical property. This includes theft of flags and other symbolic material symbols. It excludes the capture/release of living animals

When coding events according to the above categories, the following process was followed. First, an event that received a category “1: Release” was only recorded as having a second, separate “3: Damage” category if there was clear and intentional property damage that was done *in addition to* the specific acts needed for an animal's actual release. An example of property damage that would not qualify for this separate coding would be a case of an activist cutting/damaging a cage or fence to release an animal or animals. Similarly, events coded as “4: Arson” were not separately coded as “3: Damage,'' even if the arson damaged property, unless there was a separate mention of non-arson-related based property damage act that occurred alongside the arson action. Ambiguous mentions of “raids” were coded as “1: Release” if a summary designates them as occurring at (e.g.) a lab, university, or farm, but as “7: Theft” if the raid was designated as occurring in a store (e.g., a fur shop or clothing store).

**Animal (Release) Types**: For events coded as “1: Release,” coders then re-read the event's summary and coded the animal species that was (were) liberated by that liberation activity if explicitly mentioned. Because some direct-action events released multiple animal species, we include up to three animal-category variables for coding. For each relevant animal coding, coders endeavored to always code using the name of the animal in singular form (e.g., “Rabbit” not “Rabbits”).

**Data Aggregation:** Altogether the above steps produced the RALM event-level dataset described above (RALMevent.csv). Following the completed coding of RALMevent.csv, these events were next aggregated to the grid-cell year level for RALMaggregated.csv. Coded events were first subset to only include events from 1995-onward with city-level geolocation information. These retained events were merged to a static PRIO GRID template by attributing each event to its appropriate grid cell based upon (i) that event's latitude-longitude coordinates and (ii) the latitude-longitude coordinates of the retained PRIO GRID cells for the U.S. and Canada—while retaining year information. Following this, all PRIO GRID cell-indexed events were aggregated into counts of events for each year, 1995-2007. This produces the final grid-year event count aggregations discussed in RALMaggregated.csv.

The PRIO GRID-specific variables discussed in Table 4 were automatically merged to this grid-year dataset based on PRIO GRID IDs. The general socio-political event data measure outlined in Table 3 were created and added to this grid-year data in the following manner. First, the raw event data in [4] were downloaded and subset to the countries of study (i.e., U.S. and Canada) and relevant directed source-target actor combinations (i.e., civilian, government, or any domestic actor, as denoted in Table 4). The original event data in [4] were coded in an automated fashion according to the CAMEO event ontology [11] for all countries of the world from the New York Times, BBC Summary of World Broadcasts, and CIA Foreign Broadcast Information Service for the period 1945-2005. It hence provides ample country and temporal overlap for our RALM direct-action events. After subsetting the events in [4] in the manners described above, all retained events were then deduplicated across the three aforementioned news sources so as to retain only one event of a specific CAMEO event type, involving a specific source and target actor pairing, occurring in a specific country and at a specific set of latitude-longitude coordinates, and taking place on a specific calendar day. Retained events were then aggregated to a set of commonly used [[12], [13], [14]] quad categories denoting verbal and material conflict and cooperation for each directed source-target actor pairing of interest. They were then merged based on latitude-longitude coordinates to PRIO GRID's grid-cells based upon the latter's centroid latitude-longitude coordinates and aggregated to the grid-year (count) level.

The process for coding the radical environmental ‘groups’ was as follows. First, a separate section of the 30-issue No Compromise magazine titled “In the Trenches” was utilized to identify the names and mailing addresses of all active RALM groups in the US and Canada during a particular No Compromise issue's date of publication. The names and mailing addresses of each group for each issue were manually transcribed, and a spreadsheet was created with group-year-level entries that contained each corresponding group name and address. Using geonames.org, each RALM group's address was geolocated to the city-level with latitude-longitude coordinates added to the spreadsheet. Following this, each group-year entry was merged to its corresponding PRIO-GRID cell and these entries were then summed and merged so as to provide a total annual count of the number of distinct RALM groups active in each grid-cell within RALMaggregated.csv.

Finally, the three disaster variables (disastercost, disasterfatality, and disasterevent) in RALMaggregated.csv were coded as follows. First, note that [7] developed a comprehensive list of major global energy accidents for the period 1907-2007, encompassing disasters such as major oil spills and fires, nuclear plant accidents, and natural gas pipeline leaks. All such accidents for the US and Canada during the 1995-2007 period were transcribed along with their date, location of occurrence, fatalities, and estimated cost (in millions $U.S.). Latitude-longitude coordinates for each location were then manually identified and added from geonames.org. Each record was then merged to the PRIO-GRID structure for the U.S. and Canada using these coordinates and the coordinates for each retained PRIO-GRID cell's centroid. Events were then aggregated to the cell-year level by summing cost and fatality totals within each specific cell-year observation. A separate binary disaster variable was then added to provide a record of whether or not a cell-year saw at least one such disaster event.

Both datasets and a set of associated dataset creation files are publicly available at [17].

## Limitations

Note that a small number of the textual event summaries that were used for coding failed to indicate a sub-state or sub-provincestate location for a discussed event. As a result, 4.6% of all coded RALM events are missing data for the city variable in RALMevent.csv. They are hence not geocoded in RALMevent.csv nor included in RALMaggregated.csv. For similar reasons, RALMevent.csv also sees a small share of events with missing data on month (0.4% of all cases), day (12.8% of all cases), year (0.1% of all cases), and named.location (2.9% of all cases). Also note that the No Compromise-specific textual information that was utilized in coding the (RALM) groups variable included in RALMaggregated.csv was only available for 1996-2006. As a result, this variable always has missing data for 1995 and 2007. Lastly, the two datasets’ respective RALM direct-action events represent a static historical dataset for the U.S. and Canada. While expanding these data beyond 1995-2007 and North America will be challenging given available source materials, it is a future aspiration.

## Ethics Statement

The authors have read and followed the ethical requirements for publication in Data in Brief and confirm that the current work does not involve human subjects, animal experiments, or any data collected from social media platforms.

## Credit Author Statement

**Zack W. Almquist, Benjamin E. Bagozzi, and Daria Blinova:** Conceptualization, Methodology, Data Curation, Writing - Original Draft, Writing - Review & Editing, Visualization.

## Data Availability

DataverseRadical Animal Liberation Movement (RALM) Event Data (Original data).

## Footnotes

1 Note that because this website is now defunct, we have linked to a static HTML page of the original website.

2 Because this website is now defunct, we have linked to a static HTML page of the original website.
